# Harmonine, a defence compound from the harlequin ladybird, inhibits mycobacterial growth and demonstrates multi-stage antimalarial activity

**DOI:** 10.1098/rsbl.2011.0760

**Published:** 2011-09-21

**Authors:** Christian Rene Röhrich, Che Julius Ngwa, Jochen Wiesner, Henrike Schmidtberg, Thomas Degenkolb, Christian Kollewe, Rainer Fischer, Gabriele Pradel, Andreas Vilcinskas

**Affiliations:** 1 Fraunhofer Institute for Molecular Biology and Applied Ecology IME, Bioresources Project Group, Winchesterstraße 2, 35394 Gießen, Germany; 2 Institute of Phytopathology and Applied Zoology at the Interdisciplinary Research Center, Justus-Liebig University of Gießen, Heinrich-Buff-Ring 26-32, 35392 Gießen, Germany; 3 Research Center for Infectious Diseases, University of Würzburg, Josef-Schneider-Straße 2/D15, 97080 Würzburg, Germany

**Keywords:** *Harmonia axyridis*, insect immunity, harmonine, antimicrobial activity

## Abstract

The harlequin ladybird beetle *Harmonia axyridis* has been introduced in many countries as a biological control agent, but has become an invasive species threatening the biodiversity of native ladybirds. Its invasive success has been attributed to its vigorous resistance against diverse pathogens. This study demonstrates that harmonine ((17*R*,9*Z*)-1,17-diaminooctadec-9-ene), which is present in *H. axyridis* haemolymph, displays broad-spectrum antimicrobial activity that includes human pathogens. Antibacterial activity is most pronounced against fast-growing mycobacteria and *Mycobacterium tuberculosis*, and the growth of both chloroquine-sensitive and -resistant *Plasmodium falciparum* strains is inhibited. Harmonine displays gametocytocidal activity, and inhibits the exflagellation of microgametocytes and zygote formation. In an *Anopheles stephensi* mosquito feeding model, harmonine displays transmission-blocking activity.

## 1. Introduction

*Harmonia axyridis*, known as the Asian lady beetle or the harlequin ladybird, is a ladybird beetle native to continental, temperate and subtropical parts of East and Central Asia. Since the beginning of the twentieth century, this species has been introduced as a biological control agent against aphid and/or coccid pests into North America, Europe and the Soviet Union. In addition, *H. axyridis* has been commercially available as a biological control agent for greenhouses and urban ecosystems since the mid-1990s. Over the last two decades, *H. axyridis* has become an invasive species in many countries. In Europe, *H. axyridis* populations have been growing rapidly since the turn of the millennium, threatening populations of native ladybird species [1]. Its invasive success has been attributed to its enduring resistance against diverse pathogens, which allows it to outperform and therefore dominate the most abundant native European ladybirds, *Coccinella septempunctata* and *Adalia bipunctata* [2]. Besides antimicrobial peptides encoded by small genes and synthesized on ribosomes [3], many insects synthesize low-molecular mass defence compounds, or sequester such compounds from their diet. Ladybirds exude droplets of haemolymph containing deterrent alkaloids through their leg joints when threatened or attacked, a behaviour known as reflex bleeding [4]. In the present study, harmonine ((17*R*,9*Z*)-1,17-diaminooctadec-9-ene) was identified as the principal antimicrobial compound of *H. axyridis* haemolymph.

## 2. Material and methods

### (a) Origin and rearing of ladybirds

Adults of *H. axyridis* subsequently used for captive breeding were collected in and around Gießen and Ober-Mörlen, Germany. Adults of the seven-spot ladybird (*C. septempunctata*) and eggs of the two-spot ladybird (*A. bipunctata*) were obtained from Kath Biotech AG (Baruth, Germany). All ladybird species were reared in cages at 26°C and 60 per cent relative humidity under a 16 : 8 photoperiod. Bean plants (*Phaseolus vulgaris*) infested by pea aphids (*Acyrthosiphon pisum*) were provided as a food source.

### (b) Purification, structure determination and synthesis of harmonine

Haemolymph released by reflex bleeding was collected from 500 *H. axyridis* beetles. Groups of five beetles were vortexed for 10 s in 0.2 ml water in a 1.5 ml tube, and the combined liquid was heated to 95°C for 1 h and the precipitated material removed by centrifugation. The supernatant was supplemented with acetonitrile to a final concentration of 20 per cent (v/v) and passed over a strong anion exchange solid-phase extraction cartridge (ISOLUTE SAX 100 mg/3 ml, Biotage). The flow-through was loaded onto a strong cation exchange column (Mono S 5/50 GL, GE Healthcare) and eluted with a linear gradient of NaCl (0–1 M in water containing 20% acetonitrile). Fractions containing active compounds from the radial diffusion assay eluted at approximately 700 mM NaCl. After removal of excess acetonitrile by vacuum evaporation, final purification was achieved by chromatography on a reversed-phase column (Acclaim 120, C18, 3 µm, 4.6 × 150 mm; Dionex) by applying a gradient of 8–80% acetonitrile in water containing 0.1 per cent formic acid. The activity was recovered at approximately 45 per cent acetonitrile. Structure determination was performed on a micrOTOF-Q II mass spectrometer (Bruker Daltonics). Harmonine was synthesized following the protocol of Enders & Bartzen [5].

### (c) Antibacterial activity

For radial diffusion assays, beetles were homogenized in 20 per cent acetonitrile (10 µl mg^−1^ beetle weight), and 5 µl of the supernatant was applied to yeast extract and tryptone agar test plates (well diameter 3 mm) containing *Escherichia coli* DE 31. Minimal inhibitory concentration (MIC) values were determined in triplicate with 1 : 2 serial dilutions. Activity against *Mycobacterium tuberculosis* was determined using the BACTEC MGIT 960 system (Becton Dickinson).

### (d) Antimalarial activity

Synchronized cultures containing *Plasmodium falciparum* ring forms were plated in 96-well plates at a parasitaemia of 1 per cent in the presence of 1 : 2 serial dilutions of harmonine. After incubating the plates for 72 h, the viability of the parasites was assessed using the Malstat assay [6]. Gametocytocidal activity was determined by plating stage II *P. falciparum* gametocytes in triplicate in 24-well plates in the presence of harmonine [7]. The cultures were incubated with harmonine for 48 h and then for another 5 days without the compound. The numbers of stage IV and V gametocytes in 1000 red blood cells were counted. Inhibition of microgametogenesis was determined by adding harmonine to mature gametocyte cultures for 15 min at 37°C prior to activation with 100 µM xanthurenic acid. After another 15 min, the numbers of exflagellation centres were counted in 30 optical fields using a Leica DMLS microscope (400× magnification). Inhibition of zygote formation was determined by adding harmonine to mature gametocyte cultures prior to activation. The activated cultures were incubated at room temperature for 20 h. Zygotes that had subsequently formed were highlighted using anti-Pfs25 antibodies (ATCC). The numbers of zygotes were counted in 90 optical fields at 400× magnification. Transmission-blocking activity was determined by feeding female *Anopheles stephensi* mosquitoes on harmonine-containing gametocyte cultures. The mosquito midguts were dissected 12 days post-infection and inspected for oocysts by mercurochrome staining.

### (e) Antiproliferative and cytotoxic activity

Cells (HUVEC (ATCC CRL-17230), K-562 (DSMZ ACC 10) and MCF-7 (DSMZ ACC 115), Sf9 (Promega), High Five (Invitrogen)) were plated together with harmonine in 96-well plates and incubated for 72 h. Cell viability was determined using the CellTiter-Blue (Promega) or 3-(4,5-dimethylthiazol-2-yl)-2,5-diphenyltetrazolium bromide (MTT) assay. Subconfluent monolayers of HeLa cells (DSMZ ACC 57) were incubated with harmonine for 72 h before staining with methylene blue.

## 3. Results

### (a) Bioactivity guided purification of harmonine

**Figure 1.**
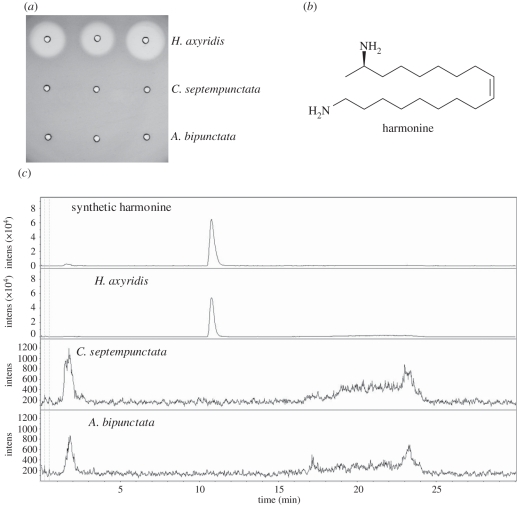
Detection of harmonine as the principal antimicrobial compound in *H. axyridis* haemolymph. (*a*) Haemolymph from three individual beetles from each of the three ladybird species were tested in radial diffusion assays against *Escherichia coli*. The activity of the *H. axyridis* samples corresponds to 499, 473 and 535 µg ml^−1^ gentamycin, respectively. (*b*) Chemical structure of harmonine. (*c*) The haemolymph samples used for the radial diffusion assays were analysed for the presence of harmonine by liquid chromatography–mass spectrometry. Extracted ion chromatograms were recorded at *m/z* 283 ± 0.5. Synthetic harmonine was analysed as a control.

In initial experiments, the antimicrobial activity of haemolymph from ladybirds was assessed using a radial diffusion assay. *Harmonia axyridis* haemolymph generated an inhibition zone that was not observed with haemolymph from *C. septempunctata* or *A. bipunctata* (figure 1*a*). The active compound was purified by collecting haemolymph released by reflex bleeding from 500 *H. axyridis* beetles. Applying the radial diffusion assay for the detection of active fractions, a purification procedure by heat denaturation, anion exchange chromatography (activity in flow-through), cation exchange chromatography and reversed-phase high-performance liquid chromatography resulted in a single compound, which was identified as harmonine ((17*R*,9*Z*)-1,17-diaminooctadec-9-ene) (figure 1*b*) by electrospray ionization quadrupole time-of-flight (ESI-Qq-TOF) mass spectrometry. Using synthetic harmonine as standard, the amount of harmonine contained in an individual *H. axyridis* beetle was determined to be 160 ± 44 µg. With an estimated haemolymph volume of 21 ± 4 µl (determined by weight loss on drying), this corresponds to a harmonine concentration of approximately 27 mM. Harmonine was neither detectable in *C. septempunctata* nor in *A. bipunctata* haemolymph (figure 1*c*).

### (b) Antibacterial activity

Synthetic harmonine displayed activity against 12 bacterial strains and the yeast *Candida albicans* with MIC values in the range between 5.5 and 354 µM (table 1). Harmonine proved to be equally active against a drug-sensitive *Staphylococcus aureus* and an multi-resistant *S. aureus* (MRSA) strain. Pronounced activity was observed against four strains of fast-growing mycobacteria (MIC values of 5.5 µM for *Mycobacterium vaccae* and 44 µM for *Mycobacterium smegmatis*, *Mycobacterium aurum* and *Mycobacterium fortuitum*). Comparable activity was also observed against *M. tuberculosis* (MIC = 44 µM).

**Table 1.** Antibacterial and anti-*Candida* activity of harmonine.

microbial strain	MIC (µM)	
	harmonine	control antibiotic^a^
*Escherichia coli* 458	89	<0.15
*Pseudomonas aeruginosa* SG137	177	0.60
*Pseudomonas aeruginosa* K799/61	354	1.2
*Bacillus subtilis* 6633	44	<0.15
*Enterococcus faecalis* 1528 (VRE)	177	2.4
*Staphylococcus aureus* 511	89	0.60
*Staphylococcus aureus* 134/93 (MRSA)	89	78
*Mycobacterium smegmatis* SG987	44	1.2
*Mycobacterium aurum* SB66	44	<0.15
*Mycobacterium fortuitum* ‘Borstel’	44	<0.15
*Mycobacterium vaccae* 10 670	5.5	0.60
*Mycobacterium tuberculosis* H37	44	0.36
*Candida albicans* C.A.	177	0.11

^a^Isoniazid and amphotericin B were used as control antibiotics for *M. tuberculosis* and *C. albicans*, respectively. Ciprofloxacin was used for all other strains.

### (c) Antimalarial activity

The growth of asexual blood stages of the protozoan parasite *P. falciparum*, which causes malaria tropica, was inhibited by harmonine with half-inhibitory concentration (IC_50_) values of 4.8 and 7.6 µM for the chloroquine-sensitive strain 3D7 and the chloroquine-resistant strain Dd2, respectively (table 2). Chloroquine treatment resulted in parasite growth inhibition with IC_50_ values of 24 (3D7) and 158 nM (Dd2), indicating the absence of cross resistance between harmonine and chloroquine.

**Table 2.** Antimalarial activity of harmonine.

*Plasmodium falciparum* strain	IC_50_ (µM)	
	harmonine	chloroquine
3D7 (asexual blood stages)	4.8 ± 0.2	0.024 ± 0.008
Dd2 (asexual blood stages)	7.6 ± 0.7	0.158 ± 0.001
NF54 (exflagellating microgametocytes)	5.8 ± 1.9	n.a.

Harmonine at 4.8 µM (IC_50_ concentration) reduced the number of *P. falciparum* NF54 gametocytes to 18 per cent compared with the 0.5 per cent dimethyl sulfoxide (DMSO) control, and all gametocytes were killed at 50 µM (electronic supplementary material, table S1). The positive control substance primaquine (IC_50_ activity on asexual blood stages = 3 µM) reduced gametocyte numbers to 42 per cent. Microgametogenesis was inhibited by harmonine with an IC_50_ value of 5.8 µM (table 2; electronic supplementary material, figure S1). Zygote formation was reduced to 17 ± 10 and 1.2 ± 0.4% at 4.8 and 50 µM, respectively.

When *A. stephensi* mosquitoes were fed on gametocyte cultures containing either 10 µM harmonine or 1 per cent DMSO, only 45 per cent of the mosquitoes were found to be infected in the harmonine group compared with 91 per cent in the control group (electronic supplementary material, table S2), indicating a significant reduction in parasite transmission (*p* < 0.05, Mann–Whitney test). The mean oocyte numbers were 1.0 ± 1.6 and 5.1 ± 5.3% in the harmonine and control groups, respectively.

### (d) Antiproliferative and cytotoxic activity

Proliferation of the human cell lines HUVEC, K-562 and MCF-7 was inhibited by harmonine with IC_50_ values of 21.3 ± 2.1, 18.5 ± 0.6 and 38.0 ± 5.6 µM, respectively. The IC_50_ values for the lepidopteran cell lines Sf9 and High Five were 57 ± 8 and 53 ± 18 µM, respectively. Cytotoxicity for HeLa cells was observed at 37.0 ± 1.7 µM, causing 50 per cent destruction of the cell monolayer.

## 4. Discussion

Harmonine had previously been isolated by Braconier *et al*. [8] based on its reactivity with Dragendorff's reagent. The same group showed that the compound acted as a feeding deterrent against the common red ant, *Myrmica rubra* [9]. Alam *et al*. [10] reported cytotoxicity against five human solid tumour cell lines and moderate inhibition of the enzymes acetylcholinesterase, prolyl endopeptidase and neuraminidase. The broad-spectrum antimicrobial activity observed in the present study demonstrates that harmonine is an important factor in beetle immunity and may explain the invasive success of *H. axyridis*. Although harmonine was less active than standard antibiotics, the MIC values were significantly below the estimated harmonine concentration in *H. axyridis* haemolymph. At antibacterial concentrations, harmonine also displayed antiproliferative and cytotoxic activity against human and lepidopteran cell lines. How *H. axyridis* is able to resist the cytotoxic potential of harmonine remains unknown. The pronounced activity of harmonine against mycobacteria together with activity against an MRSA strain is indicative for a novel mode of action, which might be exploited by the development of derivatives less toxic to human cells. At remarkably low concentrations, harmonine inhibited the growth of the malaria parasite *P. falciparum* and prevented transmission of sexual parasite stages to the mosquito. Therefore, further studies may provide a base for the development of novel anti-malarial drugs with both parasitocidal and transmission-blocking activities.
